# Ethnomedicinal Value of Antidiabetic Plants in Bangladesh: A Comprehensive Review

**DOI:** 10.3390/plants10040729

**Published:** 2021-04-08

**Authors:** Md. Masudur Rahman, Md. Josim Uddin, A. S. M. Ali Reza, Abu Montakim Tareq, Talha Bin Emran, Jesus Simal-Gandara

**Affiliations:** 1 Department of Pharmacy, International Islamic University Chittagong, Chittagong 4318, Bangladesh; josim84@yahoo.com (M.J.U.); alirezaru@gmail.com (A.S.M.A.R.); montakim0.abu@gmail.com (A.M.T.); 2 Pharmazeutisches Institut, Abteilung Pharmazeutische Biologie, Christian-Albrechts-Universität zu Kiel, Gutenbergstraße 76, 24118 Kiel, Germany; 3 Department of Pharmacy, BGC Trust University Bangladesh, Chittagong 4381, Bangladesh; 4 Nutrition and Bromatology Group, Department of Analytical and Food Chemistry, Faculty of Food Science and Technology, University of Vigo—Ourense Campus, E32004 Ourense, Spain

**Keywords:** antidiabetic plants, ethnomedicinal plants, medicinal plants, traditional plants, diabetes mellitus, antihyperglycemic

## Abstract

The use of conventional drugs to treat metabolic disorders and the pathological consequences of diabetes further increases the complications because of the side effects, and is sometimes burdensome due to relatively higher costs and occasionally painful route of administration of these drugs. Therefore, shifting to herbal medicine may be more effective, economical, have fewer side effects and might have minimal toxicity. The present review amasses a list of ethnomedicinal plants of 143 species belonging to 61 families, from distinctive domestic survey literature, reported to have been used to treat diabetes by the ethnic and local people of Bangladesh. Leaves of the medicinal plants were found leading in terms of their use, followed by fruits, whole plants, roots, seeds, bark, stems, flowers, and rhizomes. This review provides starting information leading to the search for and use of indigenous botanical resources to discover bioactive compounds for novel hypoglycemic drug development.

## 1. Introduction

Diabetes mellitus (DM) is the most prevalent, and overwhelming chronic non-communicable disease. It is a major worldwide health problem, particularly in third-world countries. Nowadays, it is considered a worldwide epidemic. DM may cause several complications, including chronic damage, dysfunction and organ failure (kidneys, heart, and blood vessels) [1]. Long-term complications of DM are cardiovascular disease [2], microangiopathy, retinopathy, nephropathy [3], and cognitive deficit [4]. According to the International Diabetes Federation (IDF) report, there are about 425 million people with diabetes in 2017, which will rise to an estimated 629 million in 2045 worldwide [5]. The estimated prevalence of DM in Bangladesh is about 11.1 million in 2000 [6]. In DM, the biguanides, sulfonylureas, alpha-glucosidase inhibitors (acarbose, miglitol, voglibose), thiazolidinediones and, meglitinides are used to lower blood glucose level as insulin and hypoglycemic agents. However, the use of antidiabetic agents is limited because of their unfavorable impacts including hypoglycemic coma and liver and kidney complications [7,8]. Hence, it is practical in the current situation to search for new and stronger phytotherapy substances with efficacy. Recently, herbal medicines have become a topic of interest, and many herbal medicines have been recommended for the treatment of diabetes. Additionally, several compounds isolated from different plant species with their mechanistic studies. The trigonelline is a major hypoglycemic alkaloid isolated from *Trigonella foenum-graecum* L., whereas steroid charantin from *Momordica charantia* L., galegine from *Galega officinalis* L., castanospermine from *Castanospermum australe* A. Cunn. and C. Fraser, panaxans A-E from *Panax ginseng* C. A. Mey., and reserpine from *Rauvolfia serpentina* (L.) Benth. ex Kurz have been isolated [9]. Hypoglycemic activity has been reported by catharanthine (alkaloid), leurosine (alkaloid), lochnerine (alkaloid), tetrahydroalstonine (yohimban alkaloid), vindoline (alkaloid ester) and vindolinine (indole alkaloid), which was isolated from *Catharanthus roseus* [10]. According to a few studies, several medicinal plants are useful in diabetes in distinct Bangladeshi local areas, divisions, and district [11,12,13,14]. Here, this review compiled a list of antidiabetic medicinal plants from the survey reports of the whole country.

## 2. Methods

We reviewed scientific articles published in journals by electronic databases (Google Scholar, PubMed, Medline, Web of Science, DOAJ, and Scopus) using specific keywords such as “medicinal plants”, “traditional plants”, “antidiabetic plants”, “antihyperglycemic plants”, “survey of antidiabetic plants”, “survey of medicinal plants”, “ethnobotanical survey”, “ethnomedicinal survey”, plus “Bangladesh”. We reviewed 96 survey articles that gave data about the utilization of therapeutic plant species that are used to treat diabetes by local communities. We utilized distributions introducing direct ethnobotanical data to prepare a list of medicinal plants to treat diabetes in Bangladesh.

## 3. Dependency in Medicinal Plants

Bangladesh is considered an excellent source for medicinal plants due to its favorable farming condition and seasonal variety. Also, Bangladesh comprises tropical forest and boggy jungle areas with bio-diverse flora. About 75% of the country’s population lives in rural territories, and almost 80% is reliant on medicinal plants for their primary healthcare whereas herbal medication is a well-known and acknowledged form of treatment [15,16]. Moreover, Bangladesh has various indigenous communities or clans, such as Chakma, Marma, Garo, Santal, Manipuri, Tripura, who still depend on their traditional or tribal medical practitioner for treatment of assorted illnesses, including, diarrhoea, infection, diabetes, cold, cough, fever, malaria, etc. These tribal practitioners have been using medicinal plants for centuries to cure completely or at least to relieve major symptoms of diseases [17].

## 4. Ethnomedicinal Use of Plants in Diabetes

**Figure 1:**
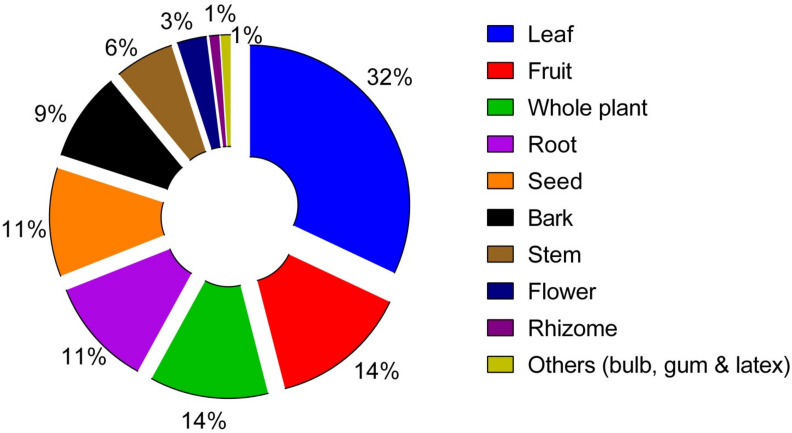
Percentage of parts of antidiabetic plants used for the treatment of diabetes in Bangladesh. Percentages were calculated as the ratio between the number of plant parts used belonging to a certain family and the total number of plants.

Treatment of hyperglycemic according to the traditional system of medicine is often easier, cheaper and cost effective due to indigenous availability of certain herbs with hypoglycemic effects [18]. A handful of ethnomedicinal surveys on medicinal plants have been accomplished from different divisions, districts, villages, and even hill tract and tribe areas of the country. A limited number of plant species have been reported to be antidiabetic. For each species, botanical name(s), family, local name(s), part(s) used, and reference(s) are presented in Table 1. Few herbal agents that possess antidiabetic properties have been cited notably in the survey, including *Azadirachta indica* A. Juss., *Centella asiatica* L. Urb., *Ficus racemose* L., *Ficus hispida* L.f., *Mangifera indica* L., *Momordica charantia* L., *Syzygium cumini* L. Skeels, *Terminalia chebula* Retz., *Coccinia grandis* L. Voigt., *Coccinia cordifolia* L. Cogn., *Aegle marmelos* L. Corrêa, *Tinospora cordifolia* Hook. F. and Thoms., *Trigonella foenum-graecum* L., *Tamarindus indica* L., *Moringa oleifera* Lam., *Kalanchoe pinnata* (Lamk.) Pers., *Bombax ceiba* L., *Cajanus cajan* L. Millsp., *Psidium guajava* L., *Clerodendrum viscosum* Vent., and *Scoparia dulcis* L. Different parts of the plants are used for antidiabetic potential such as the leaf, fruit, flower, root, bark, rhizome, bulb, latex, seed, and whole plant. Here, the leaf is the most commonly used plant part (32%) abided by the fruit (14%), whole plant (12%), root (11%), seed (11%), bark (9%), stem (6%), flower (3%), rhizome (1%), and others (bulb, gum and latex, 1%), as shown in Figure 1.

## 5. Modes of Preparation

The major modes of preparations are powder (*Syzygium cumini* L., *Azadirachta indica* A., *Momordica charantia* L., *Mikania scandens* L., *Sida cordifolia* L., *Asparagus racemosus* L., *Ficus racemosa* L.) [16,42,43,50,56,67], juice (*Cycas pectinata* B., *Cajanus cajan* L., *Ocimum tenuiflorum* L., *Moringa oleifera* Lam., *Solanum torvum* Swartz, *Coccinia grandis* L., *Stevia rebaudiana* Bertoni, *Kalanchoe pinnata* Pers., *Momordica charantia* L., *Syzygium cumini* L. [16,17,42,43,50,56,67,77], and paste (*Tinospora cordifolia* H., *Psidium guajava* L., *Nymphaea nouchali* B.) [42]. Some parts or whole plants are cooked as vegetables and eaten with meals (*Ficus hispida* L., *Momordica charantia* L., *Coccinia cordifolia* L.) [16,31,50,56,67,73] and others are also taken raw directly (*Corchorus aestuans* L., *Tamarindus indica* L., *Hibiscus schizopetalus* M.) [56,67,78,79]. Generally, whole plant or plant parts are used in the extraction of juice by soaking, crushing or boiling in water and, after that, oral administration of the juice directly or either with meals. Occasionally, plant juice or plant parts are mixed with a small amount of sugar, salt or honey before oral administration, typically to make the juice more edible and pleasant [20,120]. In a combinational medicinal plants therapy used by traditional healers (Kavirajes) for the treatment of diabetes, for example, *Azadirachta indica* A. leaves are added to the leaves of *Lawsonia inermis* L., *Costus speciosus* SM. (crêpe ginger) leaves are masticated with leaves of *Piper betle* L., and *Asteracantha longifolia* L. seeds are used in combination with *Andrographis paniculata* W. leaves, *Curculigo orchioides* G. leaves, *Ipomoea mauritiana* Jacq. leaves and fruits of *Ficus hispida* L. [49].

## 6. Antidiabetic Plant Species

The current review comprised a total of 143 plant species belong to 61 families traditionally used for the treatment of diabetes. The therapeutic plant species in the families show in Table 2. Asteraceae, and Fabaceae are characterized by nine species of each followed by Cucurbitaceae seven species; Acanthaceae and Apocynaceae six species, respectively; Lamiaceae, Poaceae and Rutaceae five species, respectively; and Combretaceae, Malvaceae and Solanaceae are represented by 4 species respectively. Triple species are found in 10 families of each and also double species are recorded in another 10 families of each. A single species in each is noted by 30 families. The review demonstrated that the common families of medicinal plant used for the treatment of diabetes in Bangladesh are Asteraceae, Fabaceae, Cucurbitaceae, Acanthaceae, Apocynaceae, Lamiaceae, Poaceae and Rutaceae. The most commonly used traditional remedies for DM are *Momordica charantia* L. (Cucurbitaceae), *Ficus racemosa* L. (Moraceae), *Syzygium cumini* L. (Myrtaceae), *Azadirachta indica* A. Juss. (Meliaceae), *Cajanus cajan* L. (Fabaceae), and *Coccinia grandis* L. J. Voigt (Cucurbitaceae).

## 7. Phytochemical and Experimental Studies of Antidiabetic Plants in Bangladesh

A common way to deal with species determination for phytochemical and pharmacological analysis is by reviewing the ethnobotanical literature [221]. Several phytochemical and in vivo studies have been executed in Bangladesh on the antidiabetic properties of traditional practitioners’ medicinal plants, divulging antidiabetic plants’ active principles. Examples of such studies are: Akter, Mahabub-Uz-Zaman, and Rahman, 2013; Al-Amin, Uddin, Rizwan, and Islam, 2013; Ali et al., 1993; Amran, Sultan, Rahman, and Rashid, 2013; Bhuyan, Rokeya, Masum, Hossain, and Mahmud, 2010; Borhanuddin, Shamsuzzoha, and Hussain, 1994b; A. Chowdhury and Biswas, 2012; A. R. Das, Mostofa, Hoque, Das, and Sarkar, 2010; Habib and Gafur, 2003; J. M. A. Hannan et al., 2003; E. Haque, Saha, Islam, and Islam, 2012; M. A. Hossain et al., 2012; Md Alamgir Hossain, Roy, Ahmed, Chowdhury, and Rashid, 2007b; M. Z. Hossain, Shibib, and Rahman, 1992; Islam et al., 2009; M. A. Islam et al., 2011; I. A. Jahan et al., 2009; Mostofa et al., 2007b; Mowl, Alauddin, Rahman, and Ahmed, 2009; Rafiq, Sherajee, Nishiyama, Sufiun, and Mostofa, 2009; Md Masudur Rahman, Hossain, Siddique, Biplab, and Uddin, 2012b; Md Mahfuzur Rahman, Sayeed, Haque, Hassan, and Islam, 2012; M. W. Rahman et al., 2005; Rokeya, Bhowmik, Khan, and Khter, 2009; M. G. Roy et al., 2010; Shahreen et al., 2012; Shibib, Khan, and Rahman, 1993; Sikder, Kaisar, Rahman, Hussain, and Rashid, 2011; Talukder, Khan, Uddin, Jahan, and Alam, 2012; Urmi et al., 2012; Zulfiker et al., 2011 [222,223,224,225,226,227,228,229,230,231,232,233,234,235,236,237,238,239,240,241,242,243,244,245,246,247,248,249,250,251,252]. These scientific studies emphasized the correlation among traditional use and the pharmacological properties of antidiabetic plants.

Various parts of the *A. augusta* plant are used in the treatment of diabetes, such as roots and leaves and bark. The methanol leaves extract of 300 mg/kg dose in alloxan-induced rat showed antidiabetic effects. In contrast, the 200 mg/kg in combination (1:1) with water extract (root and leaves) of *A. augusta* and *Azadirachta indica,* respectively, after 8 weeks exhibited significant lowering of blood sugar. In a human study, a significant blood sugar-lowering effect was observed with an alcoholic extract [253]. A significant change in body weight and decrease in blood glucose was reported by Mostofa et al., 2007 for *Catharanth roseus* leaves (1 g/kg), *Azadirachta indica* leaves (500 mg/kg), and *Allium sativum* seed (1 g/kg) aqueous extracts (14 days of treatment) [254].

According to Venkataiah et al. 2013, ethanolic roots extract of *A. ilicifolius* reported that the 200 and 400 mg/kg significantly reduced blood glucose levels in diabetic albino Wistar rat models [255], while 50, 100, 200, and 400 mg/kg doses of methanol leaves extract reported significant and dose-dependent reduction in blood glucose level of Swiss albino mice [256]. A similar result was observed by an in vitro DNSA method for aqueous, ethanol and methanol extract, whereas methanol leaves extract demonstrated highest concentration-dependent manner inhibition of α-amylase and α-glucosidase [257].

Akhtar et al., 1991 studied the aqueous and methanol extracts of the *Achyranthes aspera* whole plant demonstrated hypoglycemic activity at 2, 3, and 4 g/kg dose for alloxan-induced diabetic rabbits [258], while the ethanol leaves extract in Streptozotocin-induced rats showed a significant reduction in blood glucose level [259]. A similar result was observed in ethanol seed extract at 300 and 600 mg/kg [260].

In maceration with 80% ethanol, however, the *Adiantum capillus-veneris* extract did not demonstrate hypoglycemic activity at a dose of 25 mg/kg for mice, while the whole plant extract prepared by boiling the dried material in water was given orally to mice in same dose, glucose-induced hyperglycemia was reduced [261,262]. The alcoholic and aqueous extract exhibited a significant reduction in blood glucose level in rabbits and a DNS assay, respectively [263,264].

*A. marmelos* fruit water extract was tested in streptozotocin-induced Wistar rats at a dosage of 125 and 250 mg/kg, whereas 250 mg/kg is more efficient in lowering blood glucose [265]. Kesari et al., 2006 reported a similar result for water seed extract, whereas 100, 250 and 500 mg/kg was administered to diabetic rats [266]. An in vitro hypoglycemic activity was examined using a leaves extract of ethanol and petroleum ether in alpha-amylase inhibitory and glucose assay in yeast cells. The ethanol extract exhibited 60.2% inhibition in alpha-amylase (250 μg/mL), which was higher than petroleum ether extract [267].

*A. macrorhizome* rhizome methanol extract was used in alloxan-induced hyperglycemic mice at a single dose (250 and 500 mg/kg), whereas a substantial decrease (*p* < 0.05) in the glucose level was observed at 500 mg/kg [268].

Acetone extracts from *A. campanulatus* have been found to be possible antidiabetic agents for streptozotocin-induced Wister male diabetic rats at a dosage of 0.1% to 0.25% [269]. The corm methanol extract decreases glucose level in blood at 37.4%, with albino mice weighing 400 mg/kg, while 50, 100, and 200 mg/kg dosage also used [269,270].

Several studies reported antidiabetic effects of *A. paniculata* [271,272,273,274,275]. As of the second hour of observation, Akhtar et al. recorded 50, and 100 mg/kg water extract from *A. paniculata* leaves exhibited significantly lower glucose levels [276]. Alternatively, hot water (0.8 g/kg) and ethanol (2 g/kg) extract administration of *A. paniculata* lowered blood sugar levels in alloxan-induced diabetes rats by 46.21% and 45.13%, respectively [277]. 

The ethanol extract of *A. sativum* displayed antidiabetic effects on streptozotocin and alloxan-induced diabetic mice and rabbits by inducing insulin secretion from pancreatic parietal cells [278]. Several other studies of *A. sativum* in streptozotocin and alloxan-induced diabetes recorded which was beneficial in decreasing of the blood glucose of rats and mice [279,280]. Clinical research reported the antidiabetic effect of administering *A. sativum* pills at 900 mg/day in type-II diabetes patients [281].

In 2020, Muñiz-Ramirez et al., reported the methanol leaves extract of *A. vera* (5 mg/mL) showed 87% inhibitory activity in α-amylase enzyme, while 66% was observed in α-glucosidase enzyme [282]. *A. vera* gel (200 and 300 mg/kg) alcoholic extracts on streptozotocin-induced diabetic rats have demonstrated that they can reduce blood glucose levels without harming the subject [283]. In contrast, the administration of leaf pulp (500 mg/kg) and gel (10 mL/kg) extracts by oral administration has not been successful in another rat trial [284].

An ethanolic extract of the leaves of *A. scholaris* administration of 100, 200 and 400 mg/kg dosage by oral administration has effectively reduced blood glucose level in streptozotocin-induced diabetic rats [285]. The isolated compound from dichloromethane leaves extract, namely cycloeucalenol (a), cycloartanol (b) and lupeol (c); exposed a hypoglycemic activity at a dose of 25 mg/kg (combination of a–c) in mice [286]. In a patient based study, the leaves extract at a dose of 1, 2 and 3 g lowered the blood glucose level in a consistent manner [287].

The *Amaranthus spinosus* stems 250 and 500 mg/kg dosage [288] and leaves 200, 250, 400 and 500 mg/kg dosage [289,290] exposed antidiabetic effects in streptozotocin (STZ)-induced diabetic rats trial.

Aqueous extract and hydro-alcoholic extract from *A. mexicana* aerial parts (200 and 400 mg/kg) were reported to have hypoglycemic efficacy in alloxan and Streptozotocin-induced diabetic rats [291,292].

In 2011, Vadivelan et al. observed the blood glucose levels and fluid intake of diabetic-induced rats have substantially decreased during the oral administration of the ethanol extract of *A. racemosus*, 200 and 400 mg/kg for 21 days [293]. *A. racemosus* root was subject to α-amylase and α- glucosidase inhibitory activity in n-hexane, chloroform, ethyl acetate, and methanol, whereas less inhibitory activity of ethyl acetate and aqueous extracts was noticeable [294].

A significant reduction in plasma glucose, glycosylated hemoglobin, alanine transaminase, aspartate transaminase and total cholesterol was seen for the dose of 100, 200, and 400 mg/kg of aqueous extract of *Asteracantha longifolia* to alloxan-treated rats [295].

Shravan et al. (2011) evaluated the hypoglycemic effect of *Azadirachta indica*, whereas diabetic rat after 250 mg/kg (single and multiple dose study) treatment for 24 h and 15 days reduced creatinine, urea, lipids, triglycerides and glucose [296]. The root bark and leaves’ extracts was also effective in treating diabetes [297].

The leaf and flower portion of *B. ceiba* was extracted using various solvents, including water, 50% ethanol, and 95% ethanol, which was subjected to α-glucosidase and α-amylase inhibitory assays for antidiabetic efficacy, while the maximum effect was observed for ethanol flower extract [298]. *B. ceiba* leaf hydroalcoholic extract (200 and 400 mg/kg) showed substantial reductions in glucose levels [299].

In four separate doses of *B. pinnatum* (200, 400, 800 mg/kg and 800 mg/kg + glibenclamide 2 mg/kg), the presence of antidiabetic activity in diabetic-induced rats was shown in Aransiola et al., 2014. Their blood sugar was lower in 200 mg/kg than the other dose of aqueous extract. An 800 mg/kg aqueous extract mixture with glibenclamide (2 mg/kg), however, showed a higher efficiency than 200 mg/kg and others [300]. An anti-hyperglycemic effect on 200 and 400 mg/kg of alloxan-induced Wistar albino rats was identified [301].

*B. persicum* seed ethanol and aqueous extract decreased significantly in glucose and insulin levels at varying concentrations in diabetic rats. *B. persicum* water extract has shown protective effects on renal damage caused by diabetes in rats [302,303].

An additional study found in alloxan-induced diabetic mice that the methanol extract of *C. cajan* and *Tamarindus indica* root decreases significantly in blood glycolysis level (*p* < 0.001) in a five-day observation [192]. The antidiabetic activity of methanol extract of *C. cajan* leaves exposed a significant and dose-dependent (400 and 600 mg/kg) decrease in blood sugar of alloxan-induced diabetic rats, with the maximum effect at 4–6 h [304]. The three-dose extract of *C. indica* (100, 200, and 400 mg/kg) exhibited a significant decrease in blood glucose level [305].

*C. carandas* exhibited significant antidiabetic effects in aqueous extract (300 mg/kg), methanol fruit extract (400 mg/kg), and methanol leaves extract (50, 100 and 200 mg/kg) [306,307,308].

*C. crista* ethanol/aqueous seed extracts were subjected for antidiabetic effect in streptozotocin-induced pup models, while both ethanolic and aqueous seed extracts showed antidiabetic activity; however, aqueous *C. crista* extract had a more significant effect compared to ethanolic extract [309].

In 2008, Veeramani et al., reported antihyperglycemic effects in streptozotocin (STZ) diabetic rats by ethanolic extract of *C. halicacabum* at 50, 100, and 200 mg/kg dosage [310]. In addition, the alcoholic extract at 15, 30, and 60 mg/kg dosage significantly decrease blood glucose level in mice model [311].

A 24-week observation study on aqueous extract of *C. papaya* leaves in streptozotocin-induced diabetic rats reported reduction in fasting blood sugar, and lipid profile [312], while ethanol leaves’ extract also reported reduction in blood glucose level without any alteration of body weight [313]. In another report on ethanol leaves’ extract at a dose of 200, 400, and 600 mg/kg showed significant reduction in blood glucose level in alloxan-induced diabetic rats [314].

The *Clitoria ternatea* extract and its different fractions at 100 and 200 mg/kg dosage exposed antidiabetic effect in STZ-induced diabetic rats, while 200 mg/kg dose of ethanol and butanol exhibited significant antidiabetic and antihyperlipidemic activity [66].

*Cassia fistula* stem’s ethanolic extract significantly (*p* < 0.05) decreased blood sugar levels in alloxan-induced diabetic and glucose-induced hyperglycemic rats at 250 and 500 mg/kg, respectively. Results of glucose tolerance showed substantial improvement respectively in the dose of 250 and 500 mg/kg body weight of ethanolic extract [315].

The methanol leaves extract of *Clerodendrum viscosum* reported significant blood glucose reduction (1st to 3rd h observation) at 250 and 500 mg/kg dose in a mice model [316]. In another similar study at different doses (200 and 400 mg/kg), the extract demonstrated 25.2% and 33.3% blood glucose level reduction, respectively [317].

The ethanol *Coccinia grandis* leaves reported a non-significant hypoglycemic effect comparable to the standard drug metformin at 750 mg/kg dose [318]. Another report by Islam et al. 2014 exhibited a substantial reduction in fasting blood glucose levels from *C. grandis* and *Centella asiatica* at a dose of 3 mL/kg in both normal and therapeutic models of alloxan-induced diabetic rats [319]. In 2012, Rhaman et al., reported that the ethanolic leaves of *Centella asiatica* extract (250, 500, and 1000 mg/kg) demonstrated 32.6%, 38.8%, and 29.9% blood glucose reduction at the 3rd hourly observation, respectively, whereas no toxicity sign was observed even at 3000 mg/kg dose [226].

*Cocos nucifera* mesocarp showed (50, 100, and 200 mg/kg) significant blood glucose lowering effect with increased creatinine and glucose tolerance level for streptozotocin-induced rat [320].

The methanol and chloroform extracts of *Cuscuta reflexa* whole plants reported a significant hypoglycemic effect at the dose of 50, 100, and 200 mg/kg in glucose-induced Long-Evan rats [105]. Another report by Rath et al. 2016 exhibited that the *C. reflexa* aerial parts in methanol and aqueous extracts at the dose of 200 and 400 mg/kg showed antidiabetic effects, while the 400 mg/kg significantly reduced the blood glucose level after 3rd hour observations [106].

The chloroform extract derived from *Eclipta alba* demonstrated substantial antidiabetic efficacy in 100 type-II diabetic patients. Oral administration of *E. alba* leaf suspension (2 and 4 g/kg body weight) for 60 days leads to a significant decrease in blood glucose levels [113].

The aqueous extract derived from the seeds of *Emblica officinalis* was studied due to its antidiabetic effect in animal models. Streptozotocin-induced type-II diabetes models were considered in this regard. The results of the study reported that the doses ranging from 100–400 mg/kg body weight of this extract significantly reduced the level of blood glucose in normal rats where the reduction level was at its peak at 300 mg/kg [114].

*E. fluctuans* with partial antidyslipidemic properties in euglycemic rats and diabetic ones, appear to have a strong antihyperglycemic impact in diabetes and Cd toxicity. Twenty-one days of *E. fluctuans* extract therapy at a dosage of 200 mg/kg greatly decreased blood glucose levels in normal rats treated with both plant extract and CdCl2 (N-PCd) and diabetic treated with both plant extract and CdCl2 (DM-PCd) (*p* < 0.05) community [116].

The assessment of antidiabetic activity of *Eupatorium odoratum* leaves was conducted on male mice using alloxan with blood glucose levels >200 mg/dL. A research study has shown that the extract with dose concentrations ranging from 5–20% will reduce the blood glucose level of mice with hyperglycemia 20% more effectively [117].

*Ficus bengalensis Linn,* generally referred to as the banyan tree, is a member of the Moraceae family. Its bark is used for diabetes therapy. In this analysis, ethanol extracts from the different aerial sections of *Ficus bengalensis* Linn have been tested comparatively for their reduced blood-glucose activity. Histopathology in treatment classes for the beta-totropic function of different sections of *Ficus bengalensis* has been conducted. The ethanolic extracts of the fruit were shown to have a stronger antidiabetic influence at a dose of 120 mg/kg than the ethanol extract of the bark or root [119].

*Ficus hispida* bark ethanol extract (1.25 g/kg) shows a substantial reduction in blood glucose levels in both mild (*p* < 0.01) and diabetic (*p* < 0.001) rats. However, the blood glucose level drop was smaller than that of glibenclamide, the standard treatment [121].

The antidiabetic action of aqueous (AE) and ethanol (EE) extracts of *Ficus racemosa* was evaluated in a diabetes model induced by Streptozotocin via investigating the level of blood glucose. Treatment with AE (500 mg/kg) and EE (400 mg/kg) of *Ficus racemosa* revealed a substantial decrease (*p* < 0.05) in blood glucose levels relative to diabetic control rats [124].

*Glycosmis pentaphylla (Retz.) Correa*, a medicinal plant is widely used in Bangladesh as a herbal remedy. A study was developed for the assessment of the antihyperglycemic properties of ethanol extract of *Glycosmis pentaphylla* (GP). About 60 Swiss Albino male mice were used for this purpose (weight 20–25 g). The findings show that GP extract has a short and a week-long antihyperglycemic impact comparable to metformin HCl, a recognized and commonly used antihyperglycemic agent [125].

The effectiveness of extract from *Gymnema sylvestre* leaves was investigated in 22 type-II diabetic patients on conventional oral anti-hyperglycemic agents. GS_4_ (400 mg/day) was administered for 18–20 months as a supplement to conventional oral drugs. The supplementation of extract at a dose of 400 mg/day demonstrated a substantial reduction in blood glucose level, glycosylated plasma proteins, and glycosylated hemoglobin. These data propose that the *beta* cells can be repaired in type-II diabetic patients on *Gymnema sylvestre* extract supplementation [126].

A study was conducted in Streptozotocin-mediated diabetic rats to screen phytochemical constituents as well as the antihyperglycemic function of *Heliotropium indicum* (HI). Diabetic rats were treated with various solvent extracts of HI at a dosage of 500 mg/kg, produced substantial (*p* < 0.0001) antidiabetic activity with methanol and aqueous extracts [127].

Gayathri M. et al. 2008 evaluated the antidiabetic activity of *Hemidesmus indicus* on diabetic rats caused by streptozotocin. The results of the study concluded that aqueous extracts from the root of *H. indicus* induced significant antidiabetic activity at a dose of 500 mg/kg/day. It improves the amounts of electrolytes, hepatic microsomal protein, glucose metabolizing enzymes, and P-450 mono-oxygenase-dependent hepatic cytochrome systems at almost regular levels as well as the corresponding metabolic changes in testable induced diabetic rats [128].

Venkatesh, S. et al. conducted an experiment to find out the antidiabetic activity of *Hibiscus rosa-sinensis* flowers. *Hibiscus rosa-sinensis* ethanolic extracts at doses of 250 mg/kg and 500 mg/kg greatly decreased blood glucose levels caused by alloxan. Only a dosage of 500 mg/kg demonstrated substantial blood sugar reductions after 1 h, while the extract showed a significant drop (Pb0.05) in the level of blood glucose levels after 3 h at a dose of 250 mg/kg. A substantial decrease in blood glucose, compared to the blood glucose group treated with glibenclamide (10 mg/kg), was seen in the subacute study at a dosage of 500 mg/kg by the end of the investigation [129].

In a study, the leaves and flower extracts of *Hibiscus schizopetalus* were investigated for antihyperglycemic behaviors in alloxan-mediated diabetic rats. The hypoglycemic activity was assessed in fasting normal rats and glucose-loaded rats (100 mg/kg body weight). Body weight observations were also reported. The extracts revealed a substantial (*p* < 0.001) decrease in typical fasting rats’ blood glucose levels [130].

A study was undertaken to consider the antidiabetic efficacy of stem of *Hiptage benghalensis* where it has been shown that the extract exhibited substantial glucose absorption inhibition at a dosage of 500 mg/kg and had hypoglycemic results in Long-Evans rats of 80–200 gm [131].

The consequences of the roots and leaves of *J. adhatoda* have been studied in animals with diabetes induced by alloxan. This experiment assessed the effects of plant leaves and root extracts on blood glucose level as well as other diabetes parameters. Oral dosing of 50 and 100 mg/kg of ethanol extracts of Justicia leaves to standard and experimental diabetic rats resulted in a substantial (*p* < 0.05) decrease in blood glucose from 2 to 6 days of therapy relative to *J. adhatoda* (100 mg/kg) and glibenclamide (5 mg/kg) root extracts [132].

The antidiabetic effect in glucose-induced mice for methanol bark extract of *Lannea coromandelica* at a dose of 100, 200, and 400 mg/kg exhibited dose-dependent and significant reduction of serum-glucose levels [139].

The *Murraya koenigii* aqueous extract (200, 300, and 400 mg/kg) showed the lowering of blood glucose levels in normal as well as in diabetic rabbits after single oral administration [154]. The ethanol extract of *Mucuna pruriens* seed demonstrated a significant and dose-dependent (5, 10, 20, 30, 40, 50, and 100 mg/kg) reduction of plasma glucose level in alloxan-induced diabetic rats [155]. The stem extract of *Musa sapientum* with different doses (25, 50, and 100 mg/kg) reduced blood-glucose level in streptozotocin-induced rats, while 50 mg/kg dose was most effective [156,157]. The hot water and cold ethanol extracts of *Piper betle* leaves showed significant and dose-dependent efficacy in reducing the blood glucose level in normoglycaemic and strepozotocin-induced diabetic rats, while none of the extracts shows any toxicity sign [165].

*V. anthelmintica* exhibited significant antidiabetic effects in aqueous seeds extract (100, 200, and 500 mg/kg), and ethanol seeds extract (250, 500, and 750 mg/kg), whereas the higher showed maximum reduction in blood glucose level [203,204].

*V. rosea* exhibited significant antidiabetic effects in methanol whole plant extract at doses of 300 and 500 mg/kg in diabetic rats [321], while the alcoholic extract of leaves also reported reduction of blood glucose level [206].

The isolation of iridoid glucoside from *V. negundo* leaves were subjected for antidiabetic effect at a dose of 50 mg/kg, whereas it shows significant effectiveness in glycoprotein metabolism [208]. Idopyranose from methanol leaves’ extract at a dose of 50 mg/kg protects the pancreatic β-cells [209], while ethanolic extract (60%) was found to be a strong antidiabetic agent [210].

The methanol extract of *W. chinensis* leaf (100 and 200 mg/kg) in alloxan-induced Swiss albino diabetic mice reported antidiabetic effect, while the α-amylase inhibition assay and α-glucosidase activity exposed 48.39% and 39.37% inhibition at 500 μg/L and 10 μg/mL, respectively [211]. A significant in vitro α-amylase inhibition assay and α-glucosidase activity was observed for the isolated compound from the methanol leaves extract [212].

Ethanol *W. somnifera* roots and leaves extract at 100 and 200 mg/kg dose increase the blood glucose level while a decrease in total protein, glycogen and tissues protein [213]. Leaves and root extract showed antidiabetic activity, while the isolated compounds Withaferin A (10 μM) showed an increase glucose uptake (54%) [214].

Dosage-dependent and statistically significant antihyperglycemic activity has been shown in the *Xanthium indicum* methanol extracts in doses of 50, 100, 200, and 400 mg/kg. The higher dose (400 mg) was observed for the reduction in blood glucose level (31.2%) [217].

For antidiabetic and hypolipidemic potentials in alloxan-induced rats, *Zea mays* husk extract, and fractions (187–748 mg/kg) were used, whereas dichloromethane fraction observed the highest activity [218]. 

Antihyperglycemic and hypoglycemic behaviors were demonstrated at 200 and 400 mg/kg for aqueous extract, petroleum ether extract and the non-polysaccharide fraction of the aqueous extract of *Z. mauritiana* fruits [219]. Another study of aqueous leaves extract reported decreased hyperglycemic effects at 300 mg/kg dose [322]. The aqueous ethanol seed extract at different doses of 100, 400, and 800 mg/kg reported hypoglycemic effects [220].

## 8. Future Prospects for Antidiabetic Plant Research

According to the ethnobotanical study, almost 800 plants were reported to have antidiabetic effects [323]. Traditional plant medicines are used all over the world for diabetic presentations which may offer a natural key to uncover a critical anticipated medication for the future. For example, several plant-derived pharmaceuticals and phytotherapies presently are used by the native people of all over the world. *Galega officinalis* L. has been used since the earlier period in Europe aimed at treating symptoms associated with type-II diabetes mellitus (T2DM) [324]. It is currently accepted that its hypoglycemic and insulin-sensitizing potential is related with its guanide compound (galegine). A related compound, the biguanide metformin molecule, was later evolved and is still broadly utilized in antidiabetic treatment [325]. In addition, to treat diabetic hyperglycemia in either long or short duration, a number of natural compounds have been identified with their different mechanisms. S-methyl cysteine sulfoxide (*Allium cepa* L.) [326], lophenol (*Aloe vera* L.) [327], and gymnemic acids (*Gymnema sylvestre* R.) [328,329] contribute significant effect on insulin secreting beta cells. While S-allyl cysteine (*Allium sativum* L.) [330], insulin like protein or so called plant insulin (*Momordica charantia* L.) act as alternatives to insulin, tetrahydrocurcumin (*Curcuma longa* L.) displays its activity by modifying glucose utilization [331], and 4-hydroxyisoleucine, a novel amino acid potentiator of insulin secretion derived from *Trigonella foenum-graecum* L. [332]. Several commercially available natural products are claimed to have antidiabetic effects. It has previously been shown that *Salvia officinalis* with tea exhibited metformin-like effects [333]. *Agaricus bisporus* L. (eatable mushroom) is considered a useful nutritive aide for diabetes and showed an appreciable hypoglycemic outcome [334]. Moreover, amongst the spices, *Trigonella foenum-graecum* L. (fenugreek seeds), *Cuminum cyminum* L. (cumin seeds), *Zingiber officinale* Roscoe (ginger), *Brassica nigra* L. K. Koch (mustard), *Murraya koenigii* L. (curry leaves) and *Coriandrum sativum* L. (coriander) are reported to have hypoglycemic effects [335].

## 9. Conclusions

Bangladesh is abundant in medicinal plants that have been proved in their ethnomedicinal uses by local and ethnic people. Therefore, there is increasing evidence that old molecules are finding new therapeutic effects through better observation of traditional knowledge and clinical interpretation. Evidence-based and safe use of economical plant-derived drugs against the prevalence of diabetes may offer an enormous public health interest, particularly for developing countries like Bangladesh. Hence, we suggest an emphasis on advanced research to conduct excellent clinical studies focusing on those plants that have revealed potential antidiabetic effects.

## Figures and Tables

**Table 1:** List of ethnomedicinal plants used for the treatment of diabetes in Bangladesh.

Botanical Name	Family	Local Name(s) ^a^	Part(s) Utilized	In Vivo/In Vitro Study ^b^	Reference(s)
*Abroma augusta* L.f.	Sterculiaceae	Ulotkombol	Leaf, bark, root	Yes	[19,20,21,22,23,24]
*Abutilon indium* Sweet var.	Malvaceae	Palu-lobboi	Leaf	No	[25]
*Acanthus ilicifolius* L.	Acanthaceae	Hargoza	Root	Yes	[26]
*Achyranthes aspera* L.	Amaranthaceae	Apang, Upatlengra	Root, seed, whole plant	Yes	[11,19]
*Adiantum capillus*-*veneris* L.	Adiantaceae	Bidhayapata, Gobalelota	Seed, whole plant	Yes	[11,27]
*Aegle marmelos* L. Corrêa.	Rutaceae	Bel	Fruit, leaf	Yes	[13,28,29]
*Allium sativum* L.	Amaryllidaceae	Rosun	Root, whole plant, bulb	Yes	[11,14,29,30]
*Alocasia macrorrhizos* L. G. Don	Araceae	Mankachu	Rhizome, whole plant	Yes	[31]
*Aloe vera* L. Burm. f.	Aloaceae	Ghritokumari	Leaf	Yes	[32,33]
*Alstonia scholaris* L. R. Br.	Apocynaceae	Chaitan	Leaf	Yes	[34,35]
*Amaranthus spinosus* L.	Amaranthaceae	Katadenga	Leaf, root	Yes	[29,36,37]
*Amomum aromaticum* Roxb.	Zingiberaceae	Elach	Fruit	No	[13]
*Amorphophallus campanulatus* Blume ex Decne	Araceae	Ol	Tuber	Yes	[13]
*Andrographis paniculata* Wall. ex Nees	Acanthaceae	Kalomegh	Leaf, whole plant	Yes	[11,14,38,39,40]
*Anthocephalus chinensis* (Lam.) A. Rich. ex	Rubiaceae	Kadam	Stem, bark	No	[17,41]
*Argemone Mexicana* L.	Papaveraceae	Shialkanta	Stem	Yes	[42,43,44,45,46]
*Asparagus racemosus* L.	Asparagaceae	Sotomuli	Root, whole plant	Yes	[11,14,42,43,44,45,46,47,48]
*Asteracantha longifolia* L. Nees	Acanthaceae	Talmakhna	Seed	Yes	[49]
*Azadirachta indica* A. Juss.	Meliaceae	Neem	Bark, leaf, seed	Yes	[11,13,14,16,20,29,30,49,50,51,52,53,54]
*Bambusa tulda* Roxb.	Poaceae	Jowa bans, Mitenga	Leaf	No	[55]
*Bombax ceiba* L.	Bombacaceae	Shimul	Bark, root	Yes	[42,44,45,47,56]
*Bryophyllum pinnatum* (Lam.) Oken	Crassulaceae	Jeus	Whole plant	Yes	[57]
*Bunium persicum* Bois.	Apiaceae	Kalo jeera	Seed, whole plant	Yes	[11,14]
*Caesalpinia crista* L.	Fabaceae	Nata	Leaf	Yes	[58]
*Cajanus cajan* L. Millsp.	Fabaceae	Mehndher	Leaf, root, seed	Yes	[19,29,41,42,44,45,47,55,56,58]
*Canna indica* L.	Cannaceae	Sarbajaya, Kalaboti	Leaf, flower	No	[59]
*Cardiospermum helicacabum* L.	Sapindaceae	Phutka, Lataphutiki	Leaf, fruit	Yes	[58]
*Carica papaya* L.	Caricaceae	Pepe, Papaya	Fruit, seed	Yes	[28,60,61,62]
*Carissa carandas* L.	Apocynaceae	Koromcha	Fruit	Yes	[63]
*Cassia fistula* Linn.	Fabaceae	Sonalu, bandor lathi	Leaf, stem bark	Yes	[64]
*Cassia occidentalis* L.	Leguminosae	Sonali	Leaf, root, fruit	No	[21,28,60]
*Cassia sophera* L.	Leguminosae	Kasunda	Bark, leaf, seed	No	[25]
*Catharanthus roseus* L. G. Don	Apocynaceae	Noyontara	Leaf	Yes	[2,3,4,5,6,7,8,9,10,11,12,13,14,15,16,17,18,19,20,21,22,23,24,25,26,27,28,29,30,31,32,33,34,38,40,63,65]
*Centella asiatica* L. Urb.	Apiaceae	Thankuni	Leaf, whole plant	Yes	[11,13,14,29,30,65]
*Clitoria ternatea* L.	Fabaceae	Aparajita	Leaf	Yes	[58,66]
*Cinnamomum tamala* T. Nees and Eberm	Lauraceae	Tejpata	Leaf	Yes	[67,68,69,70]
*Cinnamomum verum* J. Presl.	Lauraceae	Daruchini	Leaf, bark	Yes	[29,71]
*Citrus aurantium* L.	Rutaceae	Jambura, Batabilebu	Fruit	Yes	[29,72]
*Citrus aurantifolia* Christm. Swingle	Rutaceae	Lebu, Kaghzilebu, Patilebu	Fruit	Yes	[29]
*Clerodendrum viscosum* Vent.	Verbenaceae	Vant, Ghetu, Baik pata	Leaf	Yes	[21,34,52,55,57]
*Coccinia cordifolia* L. Cogn.	Cucurbitaceae	Telakucha	Leaf, fruit	Yes	[15,42,44,53,55,73,74,75,76]
*Coccinia grandis* L. J. Voigt	Cucurbitaceae	Telakucha	Leaf, stem, root	Yes	[13,20,22,29,32,33,34,37,38,43,45,46,50,77,78,79,80,81,82,83,84,85,86,87,88,89,90,91,92]
*Coccinia indica* W. and A.	Cucurbitaceae	Telachuka	Fruit, leaf, root, whole	Yes	[11,14,93,94,95]
*Cocos nucifera* L.	Arecaceae	Narikel, Dab	Kernel of seed, fruit juice	Yes	[29,54]
*Colocasia esculenta* L.	Araceae	Kochu shak	Leaf	Yes	[31,96]
*Corchorus aestuans* L.	Tiliaceae	Titabhaet	Young leaf	No	[67]
*Costus speciosus* Sm.	Costaceae	Kushtha	Rhizome	Yes	[43,45,46,49,97,98]
*Cuminum cyminum* L.	Apiaceae	Jeera	Seed	Yes	[13,99]
*Curculigo orchioides* Gaertn.	Amaryllidaceae	Talmuli	Root	Yes	[63,92,100,101,102]
*Curcuma longa* L.	Zingiberaceae	Halud	Rhizome	Yes	[20,85,103,104]
*Curcuma aromatica* Salisb.	Zingiberaceae	Ban Halud	Stem	No	[52]
*Cuscuta reflexa Roxb.*	Cuscutaceae	Shornolata, Tarulata	Stem, whole plant	Yes	[30,52,64,105,106]
*Cycas pectinata Buch.-Ham*	Cycadaceae	Moniraj	Fruit, fruit stalk	No	[17]
*Cynodon dactylon* L. Pers.	Poaceae	Durba, Dubla	Leaf, whole plant	Yes	[11,14,59,73,107]
*Datura stramonium* L.	Solanaceae	Dhotura	Seed	Yes	[11,14,108,109]
*Diospyros peregrine* (Gaertn.) Gürke.	Ebenaceae	Bilati gab	Fruit	Yes	[32,110]
*Diospyros discolor* Wild.	Ebenaceae	Bilati gab	Fruit	No	[48]
*Diplazium esculentum* (Retz.) Sw.	Dryopteridaceae	Dhekishak	Root	Yes	[37,111]
*Drynaria quercifolia* (L.) J. Smith	Polypodiaceae	Pankhiraj	Stem	Yes	[21,34,112]
*Eclipta alba* L.	Asteraceae	Bringoraj, Kalokeshi	Leaf	Yes	[11,14,29,59,88,113]
*Emblica officinalis* Gaertn.	Euphorbiaceae	Amloki	Fruit, fruit pulp	Yes	[13,20,25,114]
*Enhydra fluctuans* Lour.	Asteraceae	Helencha	Leaf, stem	Yes	[34,91,115,116]
*Eupatorium odoratum* L.	Compositea	Assamlata	Leaf, flower	Yes	[51,117]
*Flacourtia indica* (Burm. f.) Merr.	Flacourtiaceae	Bouchi, Boichi	Leaf, fruit	No	[13]
*Ficus benghalensis* L.	Moraceae	Bot, Kathali Pata Bot	Leaf	Yes	[11,14,50,118,119]
*Ficus hispida* L.f.	Moraceae	Dumur, Kakdumur	Fruit, bark	Yes	[13,22,26,28,40,49,57,60,86,94,120,121]
*Ficus racemosa* L.	Moraceae	Jagadumur	Bark, fruit	Yes	[11,14,20,21,22,29,40,42,44,45,47,50,56,82,100,122,123,124]
*Geodorum densiflorum* (Lam.) Schltr.	Orchidaceae	Shonkhomuni	Whole plant	No	[22]
*Glycosmis pentaphylla* (Retz.) Corr.	Rutaceae	Ashshaora, Kawatuti	Leaf	Yes	[36,90,125]
*Gymnema sylvestre* R. Br.	Asclepiadaceae	Medhasingi, Gorshar	Whole plant	Yes	[11,126]
*Heliotropium indicum* L.	Boraginaceae	Hatisur	Leaf	Yes	[11,127]
*Hemidesmus indicus* L. R. Br.	Apocynaceae	Anantomul	Root	Yes	[11,128]
*Hibiscus rosa-sinensis* L.	Malvaceae	Jaba, Raktajaba	Flower, leaf	Yes	[25,129]
*Hibiscus schizopetalus* (Mast.) Hook. f.	Malvaceae	Shish joba	Fruit	Yes	[78,130]
*Hiptage benghalensis* (L.) Kurz.	Malphigiaceae	Madhabilata	Flower, root	Yes	[63,131]
*Hoya parasitica* Wall.	Asclepiadaceae	Chera pata	Leaf	No	[25]
*Hygrophila auriculata* (Schumach.) Heine	Acanthaceae	Kulekhara, Talmakhna	Seed	No	[86]
*Justicia adhatoda* L.	Acanthaceae	Bashok	Leaf	Yes	[20,132]
*Kalanchoe pinnata* (Lamk.) Pers.	Crassulaceae	Patharkuchi	Leaf	Yes	[42,43,44,45,46,47,56,58,73,133,134,135]
*Lagerstroemia speciosa* (L.) Pers.	Lythraceae	Jarul	Leaf, bark, seed	Yes	[11,14,21,24,136,137,138]
*Lannea coromandelica* (Houtt.) Merr.	Anacardiaceae	Jiga, Jika	Bark, root	Yes	[22,34,40,139]
*Lawsonia inermis* L.	Lythraceae	Mehedi, Mendi	Leaf	Yes	[49,51,73,120,140]
*Leonurus sibiricus* L.	Lamiaceae	Raktodrone, Guma	Leaf	No	[29]
*Mangifera indica* L.	Anacardiaceae	Aam	Seed, gum, leaf, bark	Yes	[11,13,30,48,50,54,55,73,84,141,142]
*Mikania cordata* (Burm.f.) B. L. Robinson	Asteraceae	Jarmanylata	Top of young stem, leaf, flower	Yes	[13,61,92,143,144]
*Mikania scandens* (L.) Willd.	Asteraceae	Mayalota	Leaf	Yes	[16,145]
*Mimosa pudica* L.	Fabaceae	Lojjaboti, Sada Lojjaboti	Whole plant	Yes	[11,14,146,147]
*Moghania macrophylla* (Willd.) Kuntze	Leguminosae	Blumai-kongda	Root	No	[148]
*Momordica charantia* L.	Cucurbitaceae	Korola, Usta	Fruit, leaf, whole plant	Yes	[11,13,14,15,22,29,32,34,36,40,43,44,45,47,56,57,80,83,84,85,87,133,149,150]
*Momordica cochinchinensis* (Lour.) Spreng.	Cucurbitaceae	Kakrol	Fruit	Yes	[29,151]
*Moringa oleifera* Lam.	Moringaceae	Sajna, Sajina, Khonjhon	Leaf, fruit, root	Yes	[19,22,29,38,40,45,47,79,152,153]
*Murraya koenigii* (L.) Spreng	Rutaceae	Gandhal, Girinim	Leaf	Yes	[29,61,154]
*Mucuna pruriens* (L.) DC.	Fabaceae	Alkushi	Leaf, seed	Yes	[136,155]
*Musa ornate* L.	Musaceae	Ramkola	Spadix	No	[65]
*Musa sapientum* L.	Musaceae	Kola, Aita kola	Fruit, cluster of flowers, inner trunk, young leaf	Yes	[11,13,38,40,45,156,157]
*Nymphaea nouchali* Burm.f.	Nymphaeaceae	Shapla, Sada Shapla	Leaf, whole plant, stem	Yes	[19,79,158]
*Ocimum basilicum* L.	Lamiaceae	Babui Tulshi	Leaf	Yes	[57,159,160]
*Ocimum sanctum* L.	Lamiaceae	Krisno Tulshi, Kalo Tulshi	Whole plant, Leaf, bark	Yes	[11,14,57,85,159,161]
*Ocimum tenuiflorum* L.	Lamiaceae	Tulshi	Leaf, seed	Yes	[19,162]
*Pavetta indica* L.	Rubiaceae	Kukurchura	Leaf, root	No	[58]
*Phragmites australis* (Cav.) Trin. ex Steud.	Poaceae	Nol-khagra	Whole plant	No	[60]
*Phyllanthus emblica* L.	Phyllanthaceae	Amloki	Fruit, leaf, seed, whole plant	Yes	[11,14,30,34,79,149,163,164]
*Piper betle* L.	Piperaceae	Paan	Leaf	Yes	[49,165]
*Piper cubeba* L.F.	Piperaceae	Kabab chini	Fruit	Yes	[13,166]
*Piper longum* L.	Piperaceae	Pipul, Pipla	Fruit	Yes	[13,91,167]
*Polyalthia longifolia* (Sonn.) Thwaites (PL)	Annonaceae	Debdaru	Bark	Yes	[57,85,168]
*Psidium guajava* L.	Myrtaceae	Peyara	Leaf, bark, fruit, seed	Yes	[13,19,52,60,169,170]
*Punica granatum* L.	Lythraceae	Dalim	Fruit, seed	Yes	[54,171]
*Saccharum spontaneum* L.	Poaceae	Kash, Khagra	Leaf	No	[93]
*Senna occidentalis* (L.) Link.	Fabaceae	Junjunea	Leaf	Yes	[53,172]
*Scoparia dulcis* L.	Scrophulariaceae	Bandhoney, Chinigura	Leaf, whole plant	Yes	[29,36,58,59,73,92,115,136,173]
*Sida cordifolia* L.	Malvaceae	Berela	Bark of root	Yes	[17,20,174]
*Smilax zeylanica* L.	Smilacaceae	Kumarilata	Stem	Yes	[136,175,176]
*Solanum nigrum* L.	Solanaceae	Kakmachi, Phuti begun	Leaf	Yes	[17,177,178]
*Solanum melongena* L.	Solanaceae	Begun	Fruit	Yes	[179]
*Solanum torvum* Swartz	Solanaceae	Tit baegun, Gotha begun	Leaf, root, fruit	Yes	[12,13,21,76,99,180]
*Stephania japonica* (Thunb.) Miers	Menispermaceae	Har jora	Leaf, whole plant	Yes	[100,181]
*Stevia rebaudiana* Bertoni	Asteraceae	Mistipata	Leaf	Yes	[67,182,183]
*Swietenia macrophylla* King.	Meliaceae	Mahogany	Leaf, bark	Yes	[84,91,184,185]
*Swietenia mahagoni* L. Jacq.	Meliaceae	Mahogany	Seed	Yes	[11,14,186,187,188]
*Swertia chirata* (Roxb. ex Fleming) H. Karst	Gentianaceae	Chirota	Root, Whole plant	No	[11,13,14,15]
*Syzygium aqueum* (Burm.f.) Alston	Myrtaceae	Jamrul	Fruit	Yes	[34,189]
*Syzygium cumini* L. Skeels	Myrtaceae	Jam	Leaf, bark, seed	Yes	[11,13,15,22,26,29,31,32,35,42,44,45,47,56,59,63,77,84,86,90,123,133,149,190,191]
*Tabernaemontana coronaria* Willd.	Apocynaceae	Tagar, Dudhphul	Leaf, stem bark, latex	No	[58]
*Tamarindus indica* L.	Fabaceae	Tetul	Seed, fruit	Yes	[13,17,21,24,28,41,54,80,192]
*Tagetes patula* L.	Asteraceae	Genda	Leaf	No	[75]
*Terminalia arjuna* W.and A.	Combretaceae	Arjun	Seed, bark	Yes	[11,14,20,115,120,193]
*Terminalia bellerica* (Gaertn.) Roxb.	Combretaceae	Bohera	Fruit	No	[13,17,194]
*Terminalia bellirica* L.	Combretaceae	Bohera, Jonglee bohera	Seed	Yes	[11,195]
*Terminalia chebula* Retz.	Combretaceae	Horituki	Seed, fruit, leaf	Yes	[11,13,14,34,57,196]
*Tinospora cordifolia* Hook. F. and Thoms.	Menispermaceae	Gulanchalota, Gulancha	Bark, leaf, root, whole plant, stem	Yes	[11,13,19,20,32,197]
*Tinospora crispa* (L.) Hook. F. and Thoms.	Menispermacea	Gorincha	Leaf	Yes	[22,198,199]
*Tragia involucrata* L.	Euphorbiaceae	Bichchuti	Leaf, root	Yes	[22,200]
*Trichosanthes kirilowii* Maxim.	Cucurbitaceae	Lota-mohakaal	Whole plant	Yes	[24,201]
*Trigonella foenum-graecum* L.	Fabaceae	Methi	Seed, whole plant	Yes	[11,14,202]
*Vernonia anthelmintica* Willd.	Asteraceae	Somraj	Whole plant	Yes	[11,14,203,204]
*Vinca rosea* L.	Apocynaceae	Golapi Noyontara	Leaf, stem	Yes	[11,14,205,206]
*Vitex negundo* L.	Lamiaceae	Nishinda, Samalu	Leaf	Yes	[11,14,207,208,209,210]
*Wedelia chinensis* (Osbeck) Merr.	Asteraceae	Bhimraj	Whole plant	Yes	[29,61,211,212]
*Withania somnifera* (L.) Dunal	Solanaceae	Aswagandha	Leaf, root, whole plant	Yes	[11,14,213,214]
*Xanthium indicum* Linn.	Asteraceae	Banokra, Ghagra	Leaf, root, stem, whole plant	Yes	[42,43,46,215,216,217]
*Zea mays* L.	Poaceae	Bottha	Fruit, root	Yes	[54,218]
*Zizyphus mauritiana* Lam.	Rhamnaceae	Kul, Boroi	Seed	Yes	[28,219,220]

^a^ All local name(s) are in the Bengali language. Local name(s) are adapted from survey literatures, Ethnobotanical Database of Bangladesh, and Medicinal Plants Database of Bangladesh. ^b^ The presence of antidiabetic effect (in vivo and in vitro study) was analyzed in global perspective.

**Table 2:** Presentation of the antidiabetic plant species of Bangladesh in 61 families.

Families	No. of Species	% of Species ^a^	Families	No. of Species	% of Species ^a^
Asteraceae	9	6.29	Adiantaceae	1	0.70
Fabaceae	9	6.29	Aloaceae	1	0.70
Cucurbitaceae	7	4.89	Annonaceae	1	0.70
Acanthaceae	6	4.19	Arecaceae	1	0.07
Apocynaceae	6	4.19	Asparagaceae	1	0.70
Lamiaceae	5	3.49	Bombacaceae	1	0.70
Poaceae	5	3.49	Boraginaceae	1	0.70
Rutaceae	5	3.49	Cannaceae	1	0.70
Combretaceae	4	2.79	Caricaceae	1	0.70
Malvaceae	4	2.79	Compositea	1	0.70
Solanaceae	4	2.79	Costaceae	1	0.70
Apiaceae	3	2.09	Cuscutaceae	1	0.70
Araceae	3	2.09	Cycadaceae	1	0.70
Leguminosae	3	2.09	Dryopteridaceae	1	0.70
Lythraceae	3	2.09	Flacourtiaceae	1	0.70
Meliaceae	3	2.09	Gentianaceae	1	0.70
Menispermaceae	3	2.09	Malphigiaceae	1	0.70
Moraceae	3	2.09	Moringaceae	1	0.70
Myrtaceae	3	2.09	Nymphaeaceae	1	0.70
Piperaceae	3	2.09	Orchidaceae	1	0.70
Zingiberaceae	3	2.09	Papaveraceae	1	0.70
Amaranthaceae	2	1.40	Phyllanthaceae	1	0.70
Amaryllidaceae	2	1.40	Polypodiaceae	1	0.70
Anacardiaceae	2	1.40	Rhamnaceae	1	0.70
Asclepiadaceae	2	1.40	Sapindaceae	1	0.70
Crassulaceae	2	1.40	Scrophulariaceae	1	0.70
Ebenaceae	2	1.40	Smilacaceae	1	0.70
Euphorbiaceae	2	1.40	Sterculiaceae	1	0.70
Lauraceae	2	1.40	Tiliaceae	1	0.70
Musaceae	2	1.40	Verbenaceae	1	0.70
Rubiaceae	2	1.40			

^a^ Percentages were calculated as the ratio between the number of plants belonging in a certain family and the total number of plants.

## Data Availability

All data generated or analyzed are contained within the present article.
